# Prediction of brain tissue temperature using near-infrared spectroscopy

**DOI:** 10.1117/1.NPh.4.2.021106

**Published:** 2017-06-13

**Authors:** Lisa Holper, Subhabrata Mitra, Gemma Bale, Nicola Robertson, Ilias Tachtsidis

**Affiliations:** a University of Zurich, Hospital of Psychiatry, Department of Psychiatry, Psychotherapy, and Psychosomatics, Zurich, Switzerland; b University College London and Neonatal Unit, University College London Hospitals Trust, Institute for Women’s Health, London, United Kingdom; c University College London, Biomedical Optics Research Laboratory, Department of Medical Physics and Biomedical Engineering, London, United Kingdom

**Keywords:** broadband near-infrared spectroscopy, brain temperature, prediction, calibration

## Abstract

Broadband near-infrared spectroscopy (NIRS) can provide an endogenous indicator of tissue temperature based on the temperature dependence of the water absorption spectrum. We describe a first evaluation of the calibration and prediction of brain tissue temperature obtained during hypothermia in newborn piglets (animal dataset) and rewarming in newborn infants (human dataset) based on measured body (rectal) temperature. The calibration using partial least squares regression proved to be a reliable method to predict brain tissue temperature with respect to core body temperature in the wavelength interval of 720 to 880 nm with a strong mean predictive power of $R^{2}=0.713\pm 0.157$ (animal dataset) and $R^{2}=0.798\pm 0.087$ (human dataset). In addition, we applied regression receiver operating characteristic curves for the first time to evaluate the temperature prediction, which provided an overall mean error bias between NIRS predicted brain temperature and body temperature of 0.436±0.283°C (animal dataset) and 0.162±0.149°C (human dataset). We discuss main methodological aspects, particularly the well-known aspect of over- versus underestimation between brain and body temperature, which is relevant for potential clinical applications.

## 1 Introduction

**Fig. 1:**
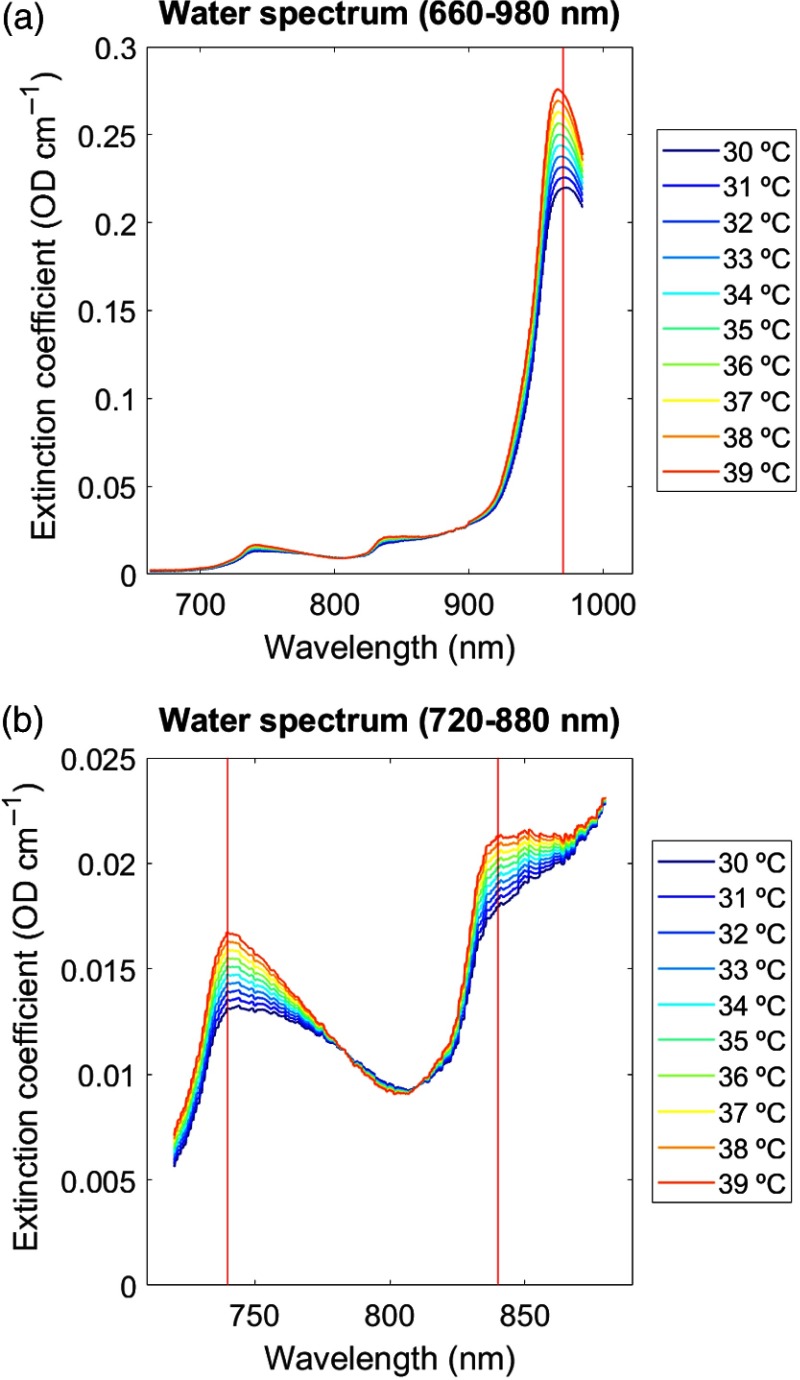
Temperature dependence of water spectrum. Extinction coefficient of pure water spectra ($H_{2}O$) simulated as a function of temperature (range 30°C to 40°C) over NIR: (a) 600 to 980 nm and (b) 720–880 nm.^7^^,^^8^ Pronounced water absorption peaks appear in the NIR around 740, 840, and 970 nm (marked by red vertical lines). Units are given in temperature (°C) with increasing extinction indicating increasing temperature (color coded from blue to red).

Near-infrared spectroscopy (NIRS) is a well-established noninvasive brain imaging method.^1^^–^^3^ NIRS is frequently used to assess changes in brain oxygenation and hemodynamics in the animal and human cerebral cortex based on the optical properties of the oxygen dependence of the hemoglobin spectrum, in addition to quantifying metabolism through measurements of the oxidation changes in cytochrome-c-oxidase.^4^ Less known is the fact that NIRS can also assess tissue temperature based on the temperature dependence of the water absorption spectrum. This unique feature of NIRS can be easily understood considering the fact that the brain is composed of ∼75% water in which temperature dependence can be assessed in the near-infrared (NIR) spectra region as previously extensively documented.^5^^–^^8^ In particular, NIRS makes use of the tissue water absorption spectrum, whose temperature-dependence changes its intermolecular hydrogen bonding with increasing light wavelength. Hence, the tissue water absorption measured by NIRS can act as an endogenous indicator of tissue temperature. Tissue in this context can be any animal or human body tissue accessible using NIRS, such as muscle or brain tissue. Previous work has shown that the most pronounced water absorption peaks obtained by NIRS occur in the NIR absorption bands around 740, 840, and 970 nm (Fig. 1). These water absorption peaks are thus thought to represent the most reliable NIRS responses to temperature and are therefore thought to allow for the prediction of tissue temperature.^7^^,^^9^^–^^11^

Generally, tissue temperature within the animal or human body is an important indicator of metabolic and thermoregulatory activity.^12^ Core body temperature, i.e., the temperature of the internal environment of the human body at usually 37°C, is typically measured at rectal, sublingual, or axillary sites. However, there might be clinical circumstances that the temperature in the brain can be different from the body’s core temperature. Therefore, only by measuring brain tissue temperature, clinically relevant temperature changes in the brain (i.e., may or may not be associated with body temperature changes) can be detected early on. Moreover, monitoring differences between brain–body temperatures can provide additional information about the intactness of the brain’s thermoregulation. Therefore, two important clinical applications traditionally attempted to measure temperature within the brain tissue. The two clinical scenarios during which brain tissue temperature measures are of tremendous benefit are therapeutic hypothermia and hyperthermia. Under hyperthermia, the local tissue temperature is raised above 40°C, e.g., in patients to eradicate tumor cells.^13^ Under hypothermia, the body temperature is lowered below 33°C, e.g., in patients suffering from traumatic brain injury^14^ or undergoing major cardiopulmonarysurgery to protect the brain against oxygen deprivation,^15^ or in newborn infants suffering from birth asphyxia as neuroprotective therapy to prevent permanent brain damage.^16^ For example, Tokutomi et al.^14^ assessed optimal temperature management during hypothermia in patients suffering from traumatic brain injury. The authors showed that intracranial pressure and cerebral perfusion pressure decreased significantly at “brain” temperatures from 37°C down to 35°C, whereas oxygen delivery and oxygen consumption decreased at “rectal” temperatures in the same °C range. However, the correlation between those brain–body measures became less significant when temperatures were less than 35°C and brain temperatures were consistently “higher” than rectal temperature by 0.5±0.3°C. These results suggest that, after traumatic brain injury, decreasing body temperature to 35°C can reduce intracranial hypertension while maintaining sufficient cerebral perfusion pressure without cardiac dysfunction or oxygen debt. This and other examples show that both hyper- and hypothermia applications require a proper assessment of the brain’s temperature in order to quickly react and prevent brain damage.

Optimal methods for brain temperature measurement should be applicable in clinical settings, even in ambulatory settings, should allow ease of handling, may be portable, and should be cost-efficient. Furthermore, to determine the efficacy of temperature treatments (e.g., hyper- or hypothermia), such devices must monitor brain temperature noninvasively and continuously throughout the treatment. So far, several methods have been evaluated for brain temperature measurements. Methods tested in more experimental research settings include proton magnetic resonance spectroscopy,^17^^–^^19^ nuclear magnetic resonance spectroscopy,^20^^–^^22^ microwave radiometry,^23^^–^^25^ and ultrasound thermometry.^26^ However, none of these methods is feasible for long-term ambulatory clinical use outside of the laboratory, nor for use in infant patients, and require large cost-intensive equipment. Other approaches are still under experimental evaluation in animals, for example, noninvasive wearable sensors to assess deep brain temperature based on skin thermal conductivity and isolation (i.e., zero-heat-flow principle),^27^ or noninvasive optical fiber-based probes based on rare-earth thermometry.^28^ Finally, there have been computational-based only approaches to estimate brain temperature changes from traditional brain recordings (i.e., such as magnetic resonance imaging) using mathematical models of brain temperature,^29^^,^^30^ that take into account the brain’s nonequilibrium thermodynamic nature between rest and functional activity.^31^ However, these approaches still remain to be tested in real practice.

Within the last decade, several attempts have also been made to evaluate NIRS for brain tissue temperature measurement. NIRS fulfills all the requirements of an optimal method for brain temperature measurement. In particular, compared to the other methods, NIRS provides applicability in clinical settings, ease of handling, portability, and cost-efficiency, for both noninvasive and continuous temperature monitoring. Early approaches using NIRS to determine brain temperature, however, were invasive, such as by applying intraparenchymal probes for intracranial temperature monitoring.^32^^,^^33^ Therefore, there was need for noninvasive NIRS techniques to monitor cerebral temperature.

So far, three publications have reported noninvasive brain temperature measurements using NIRS. The earliest work by Hollis^7^ applied continuous-wave NIRS instruments to develop, for the first time, the methodology of (nonbrain) tissue temperature based on both *in vitro* (tissue phantoms) and *in vivo* (human forearm muscle) measurements. Results of this work reported high coefficients of determination ($R^{2}>0.9$) with a mean difference between NIRS predicted temperature and thermometer measurements of 1.21°C (tissue phantoms) and 1.02°C (human forearm muscle) over a range of 28°C to 40°C. Much later, Chung et al.^11^ used broadband diffuse optical spectroscopy for (nonbrain) tissue temperature measurements *in vitro* (tissue phantoms) and *in vivo* (human forearm muscle) and also reported high coefficients of determination ($R^{2}=0.96$) with a mean temperature difference of 1.1±0.91°C over a range of 28°C to 48°C. Very recently, Bakhsheshi et al.^9^^,^^10^ used time-resolved NIRS for tissue temperature measurements *in vitro* (tissue phantoms) and *in vivo* (brain tissue in newborn piglets). The coefficients of determination ($R^{2}>0.9$) and the mean difference between the NIRS and thermometer measurements being 0.15±1.1°C (tissue phantoms) and 0.5±1.6°C (brain tissue) over a range of 32°C to 38°C again indicated very promising results.

The aim of the present analysis was twofold. We aimed to evaluate for the first time the use of (1) broadband continuous-wave NIRS in calibration and prediction of temperature in animals (newborn piglets) and (2) human brain tissue (newborn human infants).

Regarding the first aspect, broadband continuous-wave NIRS instruments are devices that acquire multiwavelength absorption spectra, typically from 650 to 1000 nm in the NIR range. Broadband NIRS provides high resolution and quantitative absorption spectra, which are essential to characterize the temperature-dependent changes associated with the water absorption features (besides the typical quantification of NIR chromophores, including oxy-, deoxyhemoglobin, lipid, and water at centimeter depths). Broadband continuous-wave NIRS is therefore much more suitable for assessing tissue temperature compared to other NIRS methods, such as discrete-wave or time-resolved NIRS.^9^^,^^10^ This differentiation is important because the latter have a reasonably lower number of wavelengths compared to broadband continuous-wave NIRS. Although it is possible to assess tissue temperature using only one, two, or three different wavelengths, such a small number of wavelengths is likely to give spurious measurements, and it is, therefore, highly advisable to use broadband NIRS.

Regarding the second aspect, broadband NIRS has so far not been evaluated in human brain tissue. We therefore aimed to evaluate the calibration and prediction of temperature in data obtained from newborn infants (human dataset). To compare the human brain tissue measures, we used data from animals, i.e., newborn piglets (animal dataset), in which NIRS has already previously been shown to successfully assess tissue temperature.^10^ The human dataset was obtained in infants undergoing hypothermia treatment and subsequent rewarming after hypoxic–ischemic encephalopathy. By contrast, the animal dataset was obtained in piglets undergoing hypothermia treatment after hypoxic–ischemic brain injury. In both datasets, core body temperature was measured rectally for reference. In order to calibrate the brain temperature prediction, we applied partial least squares regression (PLSR) following previous work.^7^ Furthermore, for the first time, we evaluated the use of regression receiver operating characteristic (RROC) curves as a very suitable graphical tool to judge the relative calibration performance in terms of over- versus underestimation between predicted brain tissue temperature and core body temperature. Based on the well-known aspect of overestimation such as reported by Tokutomi et al.,^14^ we hypothesized that the predicted brain temperature would be slightly above the measured core body temperature in our datasets.

## 2 Materials and Methods

Two datasets were obtained from the Biomedical Optics Research Laboratory, Department of Medical Physics and Biomedical Engineering, University College London, United Kingdom. The animal dataset is a subdataset from the published study by Bainbridge et al.,^34^ consisting of four newborn piglets recorded using broadband NIRS (662 to 984 nm, 322 wavelengths) during the “cooling phase” of hypothermia. The human dataset is a subdataset from the published study by Mitra et al.,^35^ consisting of four newborn infants recorded using broadband NIRS (770 to 906 nm, 136 wavelengths)^36^ during the “rewarming phase” after therapeutic hypothermia.

### 2.1 Animal Dataset

Data from four newborn piglets (PIGLET01, PIGLET02, PIGLET03, PIGLET04) included in this analysis were collected in experiments conducted at the Institute of Neurology, University College London.^34^ The newborn piglets underwent hypothermia treatment after induced hypoxic ischemia. For further information about the hypoxic ischemia protocol, the reader is referred to the publication by Bainbridge et al.^34^ During the hypothermia treatment, animals were monitored using a custom-made broadband NIRS spectrometer (662 to 984 nm, 322 wavelengths, sampling rate 0.1 Hz) in transmission mode with emitter and detector fibers placed at other side of the head, in combination with conventional systemic physiological measurements. NIRS recording was conducted from the start of the cooling phase (∼37°C) down to a target temperature of ∼33.5°C. Core body temperature was recorded using a rectal probe.

**Fig. 2:**
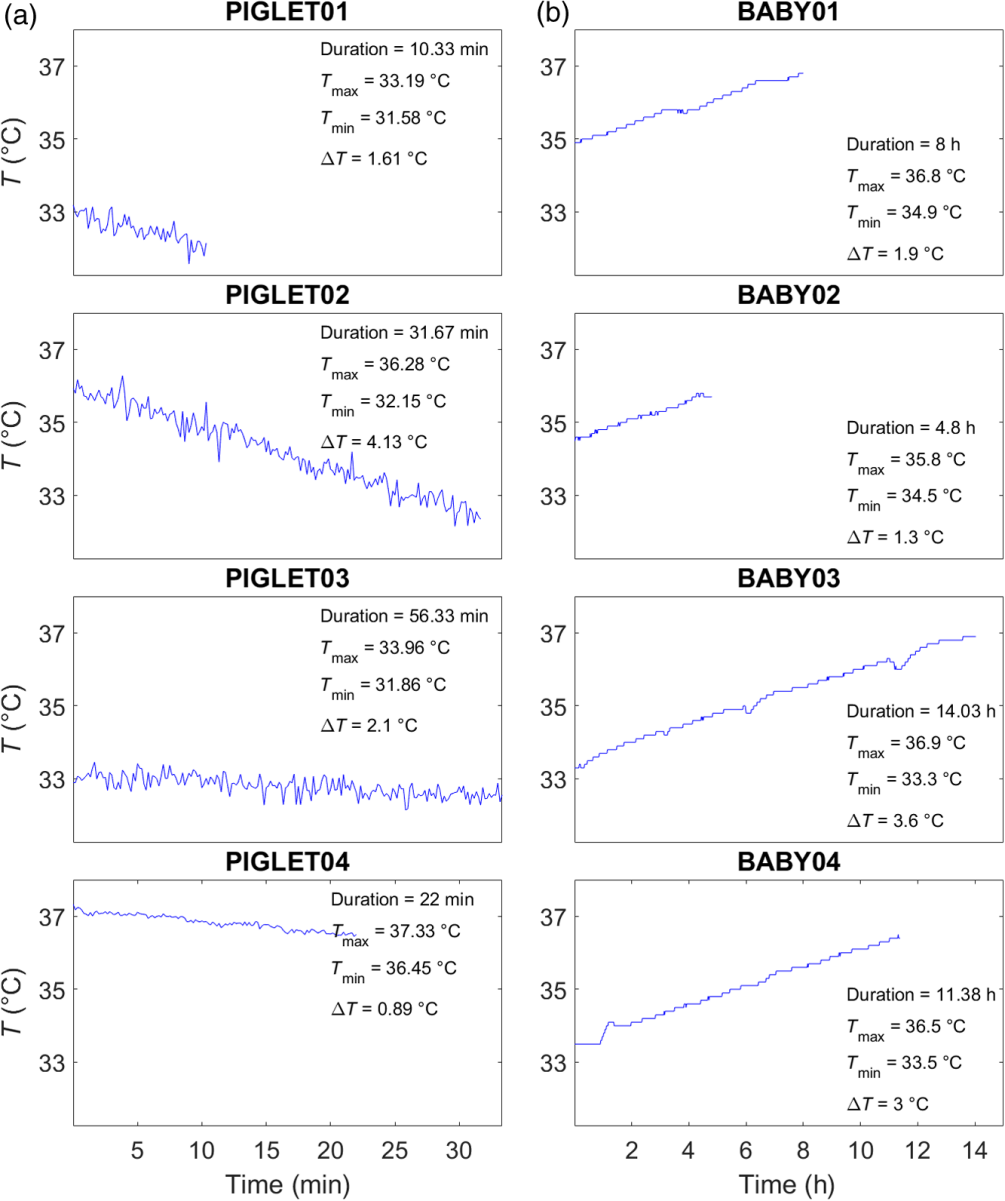
Temperature recordings. Temperature recordings (°C) based on rectal thermometer measures shown as a function of measurement duration. (a) Animal dataset. Four newborn piglets (PIGLET01, PIGLET02, PIGLET03, PIGLET04) during hypothermia treatment (cooling phase). (b) Human dataset. Four newborn infants (BABY01, BABY02, BABY03, BABY04) during hypothermia treatment (rewarming phase). The duration of the measurements is given in min (animal dataset) and h (human dataset). $T_{\max}=\text{maximum temperature measured}$, $T_{\min}=\text{minimum temperature measured}$, ΔT=temperature difference between $T_{\max}$ and $T_{\min}$.

Table 1 and Fig. 2 illustrate the temperature recordings of all animal subjects. The duration of temperature recordings from the start of the cooling phase (∼37°C) until the (expected) target temperature (∼33.5°C) ranged between ∼10 and 56 min. Note that not all animals reached the target temperature, since, in some cases, the recording had to be stopped due to unforeseen complications. There was no preprocessing done in the animal dataset.

**Table 1:** Attenuation changes. As measure of between-subject comparison of the temperature dependence, we calculated the coefficients of determination ($R^{2}$) between the attenuation changes and the corresponding 1st to 10th quantiles for the animal and human datasets. Values represent results averaged across the animal and the human dataset. See Fig. 5 for illustration.

$R^{2}$	Animal dataset			Human dataset
Absolute attenuation	740 nm	840 nm	970 nm	840 nm
	0.877	0.774	0.715	0.801
Difference attenuation	10th quantile	—	—	10th quantile
	0.963	—	—	0.577

### 2.2 Human Dataset

Data from four newborn infants (BABY01, BABY02, BABY03, BABY04) included in this analysis were collected as part of an ongoing study conducted at the University College London Hospitals NHS Foundation Trust.^35^ Ethical approval was obtained from the National Research Ethics Committee (reference: 13/LO/0225). The study examined newborn infants suffering from hypoxic ischemic encephalopathy undergoing hypothermia treatment and subsequent rewarming. During therapeutic hypothermia, core body temperature was brought down to a target temperature of ∼33.5°C and was maintained for 72 h using a servo-controlled cooling mattress machine (Tecotherm Neo, Inspiration Healthcare, United Kingdom). During the following 14-h rewarming period, the temperature was then gradually increased from 33.5°C to 37°C. During the rewarming period, infants were monitored using a custom-made broadband NIRS spectrometer (770 to 906 nm, 136 wavelengths, sampling rate 1 Hz) with two NIRS channels placed on either side of the forehead and an optode source–detector distance of 3 cm to ensure good depth penetration.^37^ Conventional systemic physiological measurements (i.e., heart rate and mean blood pressure) were also obtained. Core body temperature was recorded using a rectal temperature probe (sampling rate 0.1 Hz, later synchronized with the NIRS data sampled at 1 Hz).

Table 1 and Fig. 2 illustrate the temperature recordings of all human subjects. The duration of temperature recordings from the start of the rewarming phase (∼33.5°C) until the (expected) target temperature (∼37°C) was at least 14  h/subject, and in all subjects, the target temperature was reached. However, in the present analysis, we only considered data where concurrent measurements of systemic parameters were available; therefore, some of the recordings shown in Fig. 2 are cropped to a duration ranging between ∼4 and 14 h.

In contrast to the animal dataset, the human dataset was preprocessed as follows. First, we removed the effects of systemic changes (i.e., heart rate and mean blood pressure) from the raw NIRS data that were assumed to be independent of temperature using least-squares regression. Second, we considered the fact that the temperature in the human dataset was not raised continuously over the time course of the rewarming phase (but in 2-h steps). Therefore, in order to remove potential signal adaption in-between the temperature steps (i.e., between each 2-h period), we extracted short-time intervals after each temperature step (i.e., after each temperature increase every 2 h). In particular, we extracted 1-min intervals after each 2-h temperature increase and excluded the rest of the time series for the present analysis. From each of those extracted 1-min intervals, we then subtracted the signal baseline from the signal peak in order to obtain corresponding signal differences with respect to temperature increases. Thus, the final time series for the human dataset used in the present analysis comprised only one time point from each 2-h period.

## 3 Data Analysis

**Fig. 3:**
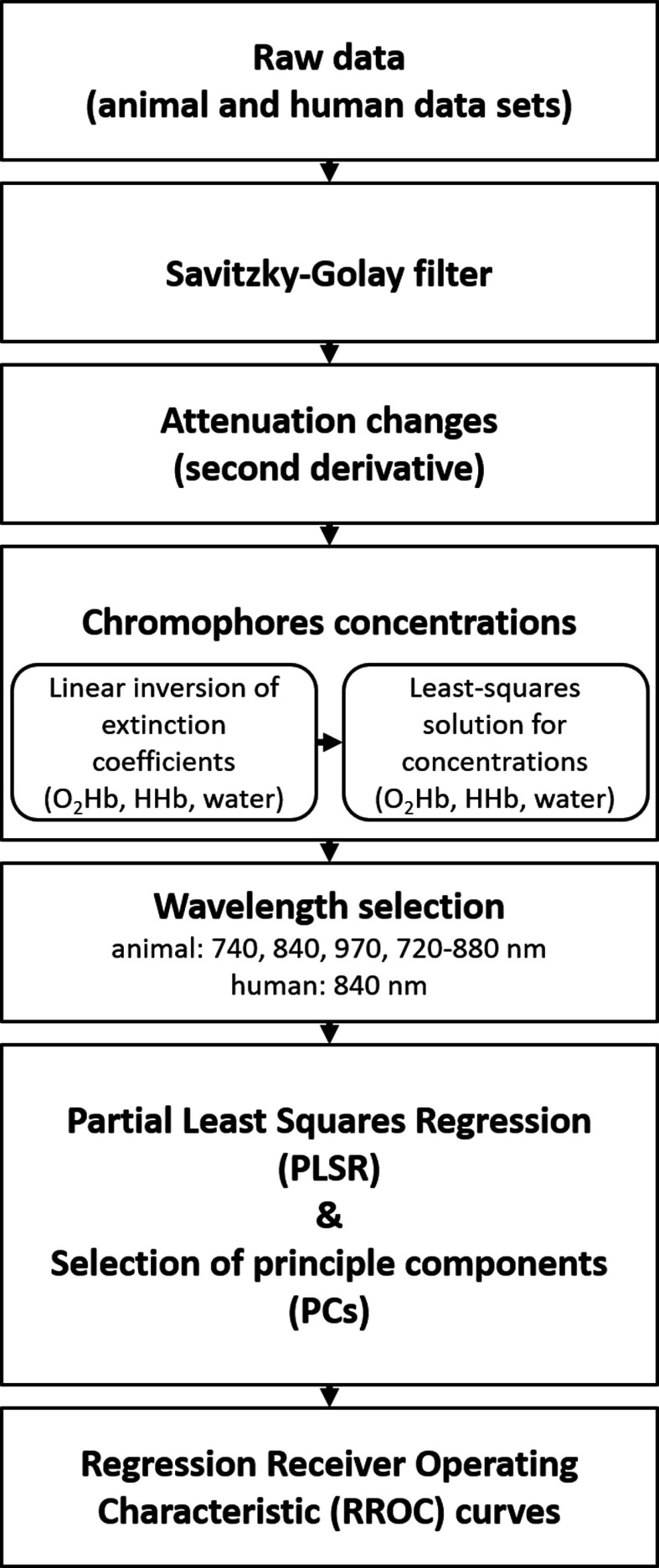
Flow chart of data analysis procedures. Raw data were smoothed using a Savitzky–Golay filter, followed by the illustration of the attenuation changes (Sec. 3.1). We then proceeded to the calculation of the chromophores concentration (Sec. 3.2), including the wavelength selection (Sec. 3.3). Subsequently, the PLSR was applied for temperature prediction, including the selection of PCs (Sec. 3.4, Fig. 4). Last, RROC curves were used to illustrate the calibration of the PLSR (Sec. 3.5). See text for further details.

Data analysis was performed using MATLAB^®^ (Version 2016a, Mathworks). A flow chart of the analysis procedure (Fig. 3) schematically illustrates (1) the demonstration of temperature-dependent attenuation changes, (2) the assessment of chromophores concentrations, (3) the calibration method in terms of PLSR for prediction of tissue temperature, and (4) the evaluation of calibration performance using RROC curves.

### 3.1 Attenuation Changes

We first illustrated the temperature dependence of the raw attenuation data in relation to wavelength. For a clearer display, the attenuation data of both datasets were smoothed using a Savitzsky–Golay filter (60-point) and binned into quantiles. In particular, two approaches were used to bin the data.

1. Temperature-binned attenuation: First, the attenuation data were binned such that the resulting plots represented the 1st to 10th quantiles of the corresponding temperature changes. Note that these quantiles covered different amounts of data points per animal/human subjects depending on the individual total time duration of the temperature recording. Using the temperature-binned attenuation data, the “absolute attenuation” was then illustrated across all animal/human subjects.
2. Wavelength-binned attenuation: Second, the attenuation data were binned such that the resulting plots represented the 1st to 10th quantiles of the corresponding wavelength range with respect to onset temperature. Using these wavelength-binned attenuation data, the “difference attenuation” was illustrated across all animal/human subjects.

### 3.2 Chromophores Concentrations

We then calculated the concentrations of the three tissue chromophores [oxyhemoglobin ($O_{2}\mathrm{Hb}$), deoxyhemoglobin (HHb), and water ($H_{2}O$)], based on the technique of second-derivative spectroscopy.^8^^,^^38^ The chromophore concentrations were calculated from the measured changes in broadband NIR light attenuation using the modified Beer–Lambert law based on the UCLn algorithm.^39^ The algorithm divides the second derivative of the tissue attenuation spectra (ΔA) by the optical pathlength, followed by a least-square regression:

$$[\begin{array}{c} \Delta [O_{2}\mathrm{Hb}]\\ \Delta [\mathrm{HHb}]\\ \Delta [H_{2}O] \end{array}]=\frac{1}{\text{pathlength}}{\left[\begin{array}{ccc} \epsilon O_{2}\mathrm{Hb}(\lambda_{1}) & \epsilon \mathrm{HHb}(\lambda_{1}) & \epsilon H_{2}O(\lambda_{1})\\ \epsilon O_{2}\mathrm{Hb}\left(\lambda_{2}\right) & \epsilon \mathrm{HHb}(\lambda_{2}) & \epsilon H_{2}O(\lambda_{2})\\ \vdots & \vdots & \vdots \\ \epsilon O_{2}\mathrm{Hb}(\lambda_{n}) & \epsilon \mathrm{HHb}(\lambda_{n}) & \epsilon H_{2}O(\lambda_{n}) \end{array}\right]}^{-1}*[\begin{array}{c} \Delta A(\lambda_{1})\\ \Delta A(\lambda_{2})\\ \vdots \\ \Delta A(\lambda_{n}) \end{array}],$$ (1)

where a linear inversion operator of the extinction coefficients (ϵ) of the three tissue chromophores was used to recover their concentrations ($\Delta [O_{2}\mathrm{Hb}]$, Δ[HHb], $\Delta [H_{2}O]$). Total hemoglobin Δ[tHb] was derived as the sum of $\Delta [O_{2}\mathrm{Hb}]$ and Δ[HHb]. The optical pathlength was derived from the product of the distance between the optodes (3 cm) and the differential pathlength factor (DPF), which has been previously measured as 4.99 (±9%) on the newborn head, corrected for the wavelength dependency of the DPF.^36^ The extinction spectra were obtained from the University College London, Engineering Faculty, Department of Medical Physics & Biomedical Engineering, London.^40^

### 3.3 Wavelength Selection

**Fig. 4:**
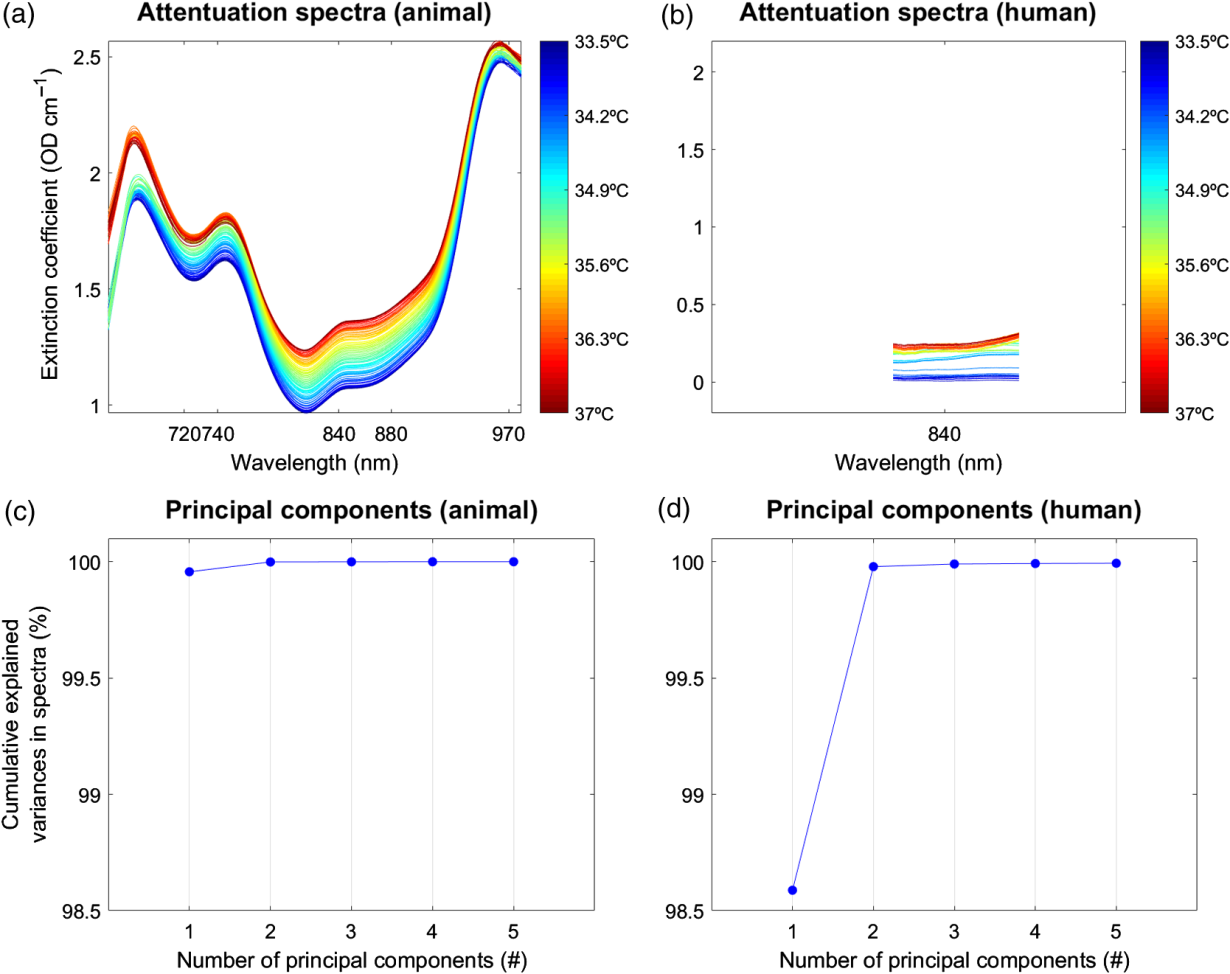
PLSR and PCs. The PLSR procedure described in Sec. 3.4 is shown for example data (PIGLET02, BABY01). Temperature is shown on the y-axis. Selected wavelengths for the animal dataset (i.e., 740, 840, 970, and 720 to 880 nm) and the human dataset (i.e., only 840 nm) are shown on the x-axis. Note that for better comparison of the two datasets, the x-axes representing the wavelengths are shown for the whole range from 660 to 100 nm; therefore, the human dataset covers only a small range. At the end of the PLSR procedure, the PCs are shown for an exemplary number of n=5 (note that we used up to 10 PCs for analysis), for the variance explained in the above spectra. See text for further details. (a) Attenuation spectra (animal), (b) attenuation spectra (human), (c) principal components (animal), and (d) principal components (human).

Broadband NIRS offers multiwavelength absorption spectra. For temperature prediction, pervious work has shown that the wavelength interval from 720 to 880 nm provides the best calibration results.^7^ However, since the animal (662 to 984 nm, 322 wavelengths) and the human (770 to 906 nm, 136 wavelengths) dataset comprised different ranges of wavelength, the data selection for the following analysis was different (Fig. 4). For the animal dataset, four wavelengths, i.e., 740 nm, 840 nm, 970 nm, and 720–880 nm, were selected. For the human dataset, only the wavelength 840 nm could be selected.

### 3.4 Temperature Calibration and Prediction

For the temperature prediction, we applied PLSR^41^ (Fig. 4) to calibrate the attenuation spectra, as previously described by Hollis,^7^ and implemented in “Toolbox for multivariate calibration techniques”^42^ for MATLAB. The PLSR calibration allows one to predict temperature without any prior knowledge of the measured temperature. PLSR is a method to model a response variable (i.e., measured temperature) in the presence of a large number of highly noisy, correlated, or even collinear, predictor variables (i.e., wavelengths). Based on these original data, PLSR then constructs new predictor variables, known as principal components (PCs), as linear combinations of the original predictor variables. The PCs give an estimation of the explained variance of the predictor variables.

In the first step of the PLSR calibration, a simulated dataset^7^^,^^10^ was built based on the temperature-dependent extinction coefficient of the pure water spectrum that comprised the independent variables (i.e., absorptions at many wavelengths) at temperatures ranging from 30°C to 40°C (Fig. 1). The simulated dataset was then compressed using PLSR to a smaller number of variables, with the resulting matrices known as the scores and the loadings, for each PCs. Subsequently, leave-one-out cross-validation was performed in order to find the minimum number of PCs for best performance (Fig. 4). We considered up to 10 PCs as implemented in a 10-fold cross-validation to choose the number of components that minimized the expected error when predicting the response from future observations on the predictor variables in the PLSR.

In the second stage of the PLSR calibration, a linear relationship between the scores and the dependent variable, i.e., the simulated temperature, was established in order to obtain a calibration vector. This calibration vector was then used to calibrate each of the individual data (of the animal and human datasets) for temperate prediction. Finally, the predicted temperature for each individual dataset was obtained by multiplying the attenuation data by the calibration vector. To correct for baseline differences between measured and predicted temperature, a constant (i.e., the baseline measured temperature, e.g., 37°C) was added to the initial predicted temperature.^7^^,^^10^

To evaluate the calibration of the PLSR, we assessed the fit between the measured (rectal) temperature and the predicted (brain) temperatures using the coefficients of determination ($R^{2}$), the mean absolute error (MAE), and the mean squared error (MSE) that were computed for each individual subject. To statistically test the calibration results of the PLSR, repeated-measures ANOVA for the coefficients of determination ($R^{2}$) as the dependent variable was applied using the within-subject factor “wavelength” and the between-subject factor “number of PCs.”. The corresponding partial eta-squared ($\eta_{p}^{2}$) of each factor was reported to determine the effect size on a significance level p<0.05. The Bonferroni correction was used to control for multiple comparisons.

Note that in a preanalysis, we tested other calibration approaches, as suggested by Hollis.^7^ The preanalysis revealed that the PLSR was superior in terms of temperature prediction compared to classical least squares, inverse least squares, and principal component regression (Table 2). In the present work, we therefore concentrated only on PLSR.

**Table 2:** Temperature prediction (preanalysis). Coefficients of determination ($R^{2}$) illustrating the predictive power of temperature prediction based on classical least squares (CLS), inverse least squares (ILS), and principal component regression (PCR) (one to three PCs), for the animal and the human datasets. Values represent results averaged across the animal and the human dataset.

	Animal dataset				Human dataset
	740 nm	840 nm	970 nm	720 to 880 nm	840 nm
	CLS				CLS
$R^{2}$	0.231	0.299	0.034	0.383	0.670
	ILS				ILS
$R^{2}$	0.538	0.609	0.077	0.684	0.697
	PCR				PCR
$R^{2}$ (one PC)	0.593	0.688	0.112	0.690	0.788
$R^{2}$ (two PCs)	0.593	0.689	0.111	0.706	0.789
$R^{2}$ (three PCs)	0.590	0.684	0.111	0.706	0.790

### 3.5 Temperature regression receiver operating characteristic

To graphically illustrate the calibration of the PLSR, we applied RROC curves,^43^ which we implemented in MATLAB^®^. RROCs have been introduced as an equivalent to receiver operating characteristic (ROC) curves for regression analyses. For the present purpose, we made use of three RROC evaluation metrics that we found to be very suitable for a between-subject comparison of the calibrated temperature prediction:

1. RROCs determine the “bias” of the predictions based on over- versus underestimation derived from asymmetric loss functions. In other words, RROCs graphically illustrate whether or not a given temperature prediction has a tendency to be under- or overestimated. Numerically, the over- and underestimation is reflected by the mean error bias (MEB) parameter, which is essentially an equivalent to the mean temperature difference used in the previous work.^7^^,^^10^^,^^11^
  MEB=predicted temperature−measured temperature. (2)
2. RROCs provide the area over the RROC curve (AOC) as an equivalent to the error variance of the regression. Thus, the AOC of a RROC serves as a measure of the expected variance for a regression model.
3. Similar to ROC curves, a RROC curve dominates another if it is always above the other. Based on this, RROC plots illustrate subject-specific (or method-specific) dominance intervals.

Linear relationships between the evaluation metrics of the temperature calibration (i.e., $R^{2}$, MAE, and MSE) and the RROCs (i.e., MEB and AOC) were assessed using Pearson product-moment correlation. Results were ported on a significance level p<0.05.

## 4 Results

As mentioned above, the animal dataset comprised a larger wavelength range (662 to 984 nm, 322 wavelengths) compared to the human dataset (770 to 906 nm, 136 wavelengths). Therefore, results of the animal dataset are illustrated based on four wavelengths, i.e., 740, 840, 970, and 720 to 880 nm, whereas the human dataset is illustrated only for the wavelength 840 nm. As mentioned earlier, these specific wavelengths (740, 840, 970, and 720 to 880 nm) were used since they have been shown to represent the most reliable water absorption peaks in the NIR range, with the wavelength interval 720 to 880 nm providing the best calibration results (Fig. 1).^7^^,^^9^^–^^11^

### 4.1 Attenuation Changes

**Fig. 5:**
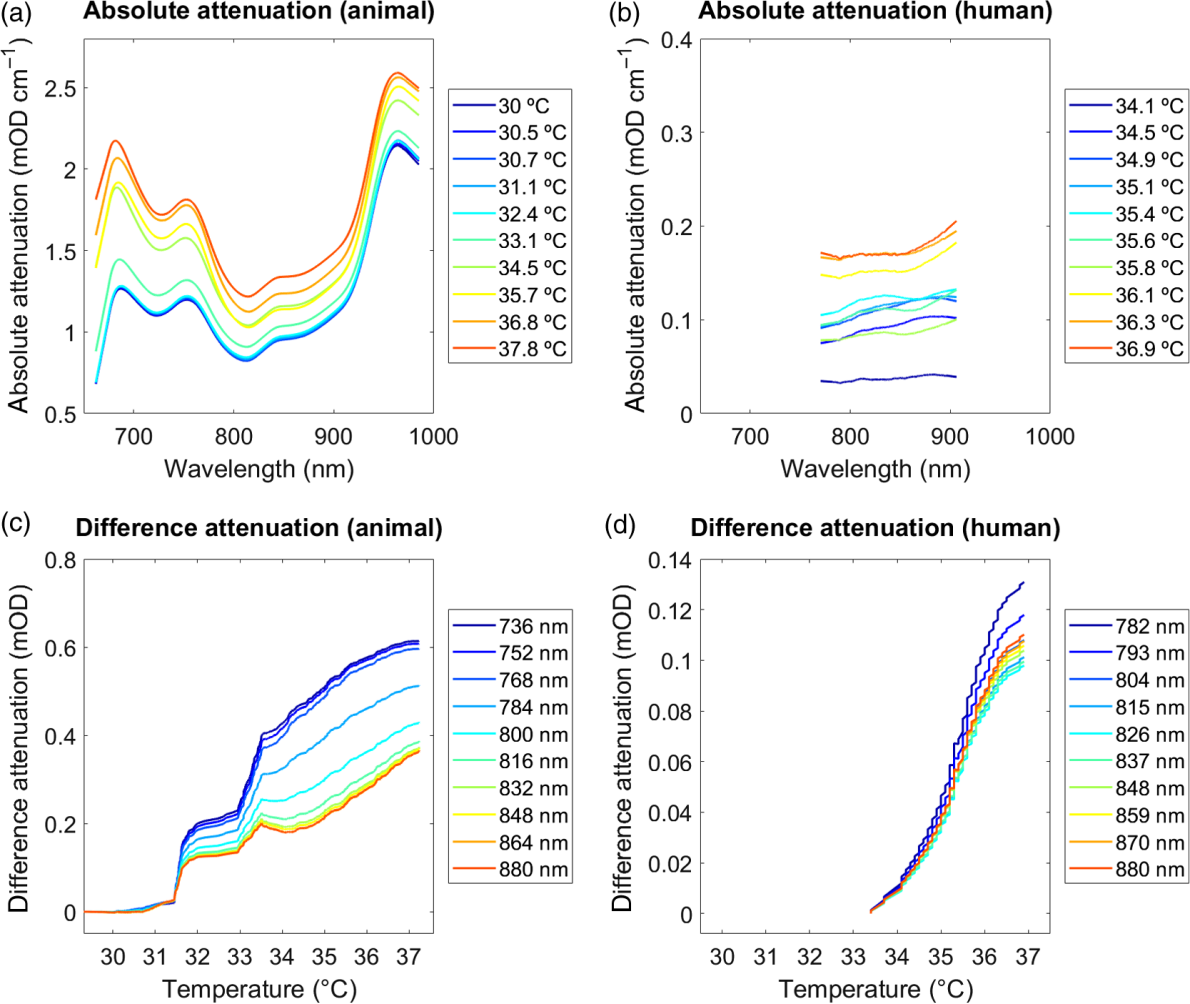
Attenuation changes. (a) and (b) Absolute attenuation. Absolute changes in attenuation over wavelengths 662 to 984 nm (animal dataset) and 770 to 906 nm (human dataset). Units are given in temperature (°C). Plots illustrate data across all animals. (b) Difference attenuation. Difference attenuation averaged over wavelengths 720 to 880 nm (animal dataset) and 770 to 880 nm (human dataset), displayed as a function of decreasing tissue temperature. Units are given in wavelengths (nm). See Table 1 for statistics.

The temperature dependence of the attenuation data is illustrated in Fig. 5.

1. Temperature-binned attenuation: The illustration of the “absolute attenuation” demonstrated the temperature dependence of the attenuation changes as a function of wavelengths based on the 1st to 10th quantiles of the corresponding temperature changes. The plot showed that attenuation at all wavelengths increased significantly with increasing temperature. In other words, attenuation decreased significantly during tissue cooling (animal dataset) and increased during tissue rewarming (human dataset).
2. Wavelength-binned attenuation: The illustration of the “difference attenuation” demonstrated the temperature dependence of the attenuation changes as a function of temperature based on the 1st to 10th quantiles of the corresponding wavelengths. Here, attenuation changes were shown with respect to that attenuation measured at the minimum temperature. This plot confirmed that attenuation increased significantly at all wavelengths with increasing temperature.

As the measure of a between-subject comparison of the temperature-dependence, we calculated the coefficients of determination ($R^{2}$) between the attenuation changes and the corresponding 1st to 10th quantiles for each single subject. Table 1 lists the corresponding $R^{2}$ averaged across all subjects, demonstrating an overall strong linear relationship between attenuation changes and temperature ($R^{2}\;\text{range}=0.577$ to 0.963).

### 4.2 Chromophores Concentration

To estimate the chromophores concentrations ($O_{2}\mathrm{Hb}$, HHb, and $H_{2}O$), we fitted the attenuation spectra to the corresponding specific absorption/extinction coefficients. Table 3 shows the coefficients of determination ($R^{2}$) assessing the relationship between $\Delta [H_{2}O]/\Delta [\mathrm{tHb}]$ and the measured temperature as determined by the least-square fits.

**Table 3:** Chromophores concentration. Coefficients of determination ($R^{2}$) assessing the relationship between the concentration changes (Δ[tHb], $\Delta [H_{2}O]$) and the measured temperature for the animal and the human datasets. Values represent results averaged across the animal and the human datasets.

	Animal dataset				Human dataset
	740 nm	840 nm	970 nm	720 to 880 nm	840 nm
$\Delta [H_{2}O]$$R^{2}$	0.130	0.132	0.107	0.601	0.660
Δ[tHb]$R^{2}$	0.183	0.636	0.132	0.676	0.293

Results showed that the water concentration $\Delta [H_{2}O]$ increased with decreasing temperature over the temperature range from 32°C to 37°C. Following the interpretation by Hollis,^7^ this may be due to increased photon penetration depth with decreasing temperature (decrease in the optical coefficients), because the light would travel through a higher proportion of tissue, which also contains a greater concentration of water.^8^ By contrast, the total hemoglobin content Δ[tHb] decreased with decreasing temperature from 32°C to 37°C. Again following the interpretation by Hollis,^7^ this might be due to physiological effects of the tissue cooling, such as the well-known shift in the hemoglobin oxygen dissociation, the increased absorption by both $O_{2}\mathrm{Hb}$ and HHb, as well as vascular thermoregulatory responses, which may result in a decrease of the total amount of hemoglobin within the region probed by the light. Note that for the following temperature prediction, only the chromophores concentrations of water ($H_{2}O$) were used, whereas those of the hemoglobin ($O_{2}\mathrm{Hb}$, HHb) were not required but served as an internal control. To illustrate this fact, we performed a simulation that is described in Fig. 8.

### 4.3 Temperature Calibration and Prediction

The brain tissue temperature was predicted based on the temperature coefficient from the pure water absorption spectra using cross-validated PLSR. Leave-one-out cross-validation was performed in order to find the minimum number of PCs for best performance (we considered up to 10 PCs; Fig. 3). For the final predictions, we calibrated the temperature using one to three PCs for the wavelengths 740, 840, 970, and 720 to 880 nm for the animal dataset and 840 nm for the human dataset. Note that the inclusion of more than three PCs did not improve the predictions.

**Fig. 6:**
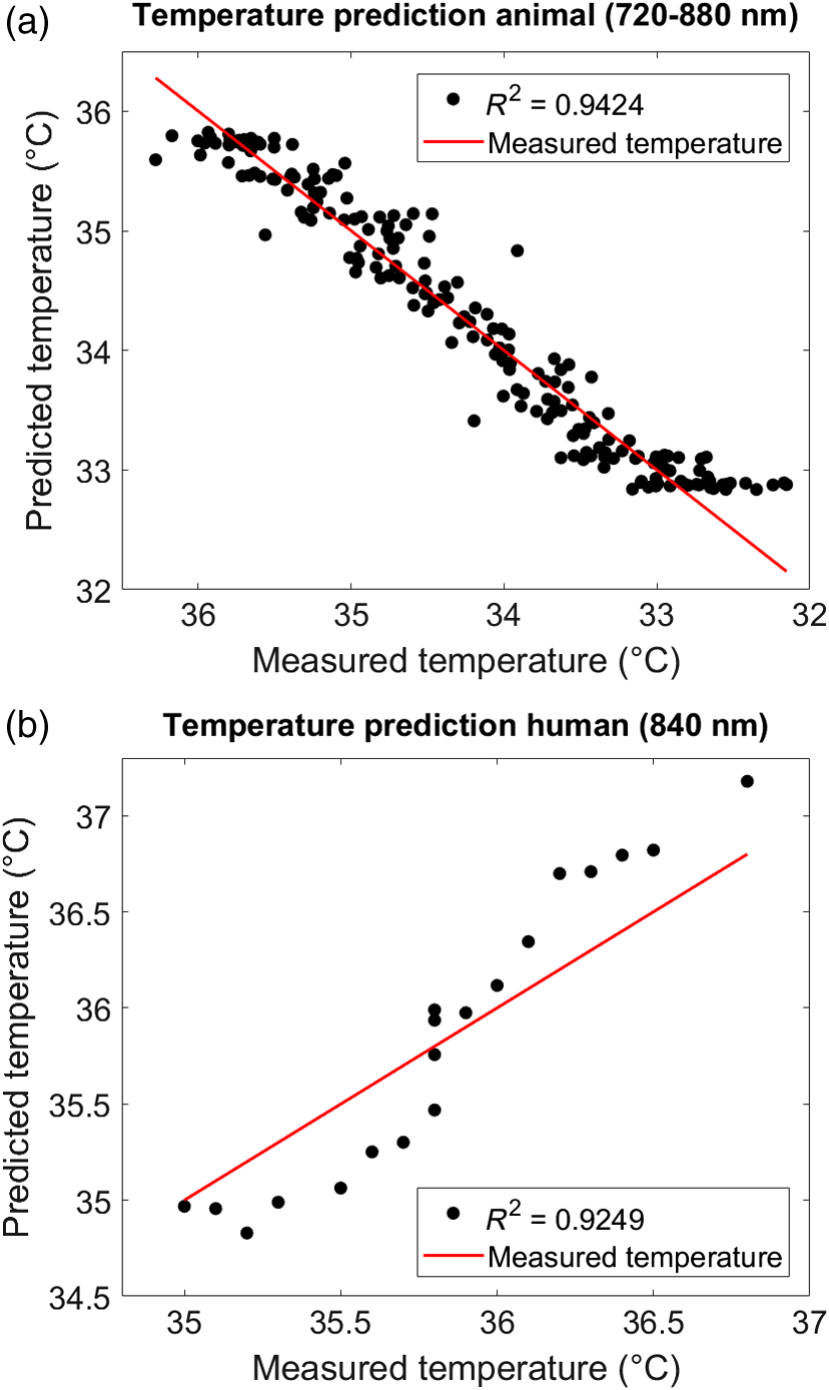
Temperature prediction. Predicted (brain) versus measured (rectal) temperature based on cross-validated PLSR. Plots exemplarily illustrate (a) the animal dataset (PIGLET02) during the cooling phase (wavelength interval 720 to 880 nm with three PCs) and (b) the human dataset (BABY01) during the rewarming phase (wavelength 840 nm with three PCs). See Table 4 for statistics.

Figure 6 shows the plots between the measured (rectal) temperature and the predicted (brain) tissue temperature for two exemplary subjects (animal dataset: PIGLET02; human dataset: BABY01) based on the best performing combinations (animal dataset: wavelength interval 720 to 880 nm with three PCs; human dataset: wavelength 840 nm with three PCs). To quantify the relationship between the measured temperature and predicted temperature, the coefficients of determination ($R^{2}$) were calculated. The mean (±STD) predictive power averaged over all individuals of the animal dataset was $R^{2}=0.713\pm 0.157$ (720 to 880 nm), and the mean predictive power averaged over all individuals of the human dataset was $R^{2}=0.798\pm 0.087$ (840 nm). Results of the summary statistics across all wavelength intervals and PCs are listed in Table 4.

**Table 4:** Temperature prediction. Coefficients of determination ($R^{2}$) illustrating the predictive power of temperature prediction based on cross-validated PLSR (one to three PCs), averaged across the animal and the human datasets. See Fig. 6 for illustration.

	Animal dataset				Human dataset
	740 nm	840 nm	970 nm	720 to 880 nm	840 nm
$R^{2}$ (one PC)	0.599	0.695	0.113	0.697	0.796
$R^{2}$ (two PCs)	0.599	0.696	0.112	0.713	0.797
$R^{2}$ (three PCs)	0.596	0.691	0.112	0.713	0.798

To statistically assess the calibration results on the group-level, repeated-measures ANOVA was performed with the coefficients of determination ($R^{2}$) as dependent variable. In the animal dataset, ANOVA revealed a significant effect of the within-subject factor “wavelength” (F=459.437, p<0.001, $\eta_{p}^{2}=0.981$). Postdoc pairwise comparison indicated significant smaller $R^{2}$ for the wavelength 970 nm compared to 740, 840, and 720 to 880 nm (all p<0.001); however, there was no effect of the between-subject factor “number of PCs” (F=0.001, p=0.999, $\eta_{p}^{2}=0.000$). In the human dataset, ANOVA revealed no effect of the between-subject factor “number of PCs” (F=0.001, p=0.999, $\eta_{p}^{2}=0.000$; the effect of “wavelength” was not present and therefore not assessed).

### 4.4 Temperature Regression Receiver Operating Characteristic

**Fig. 7:**
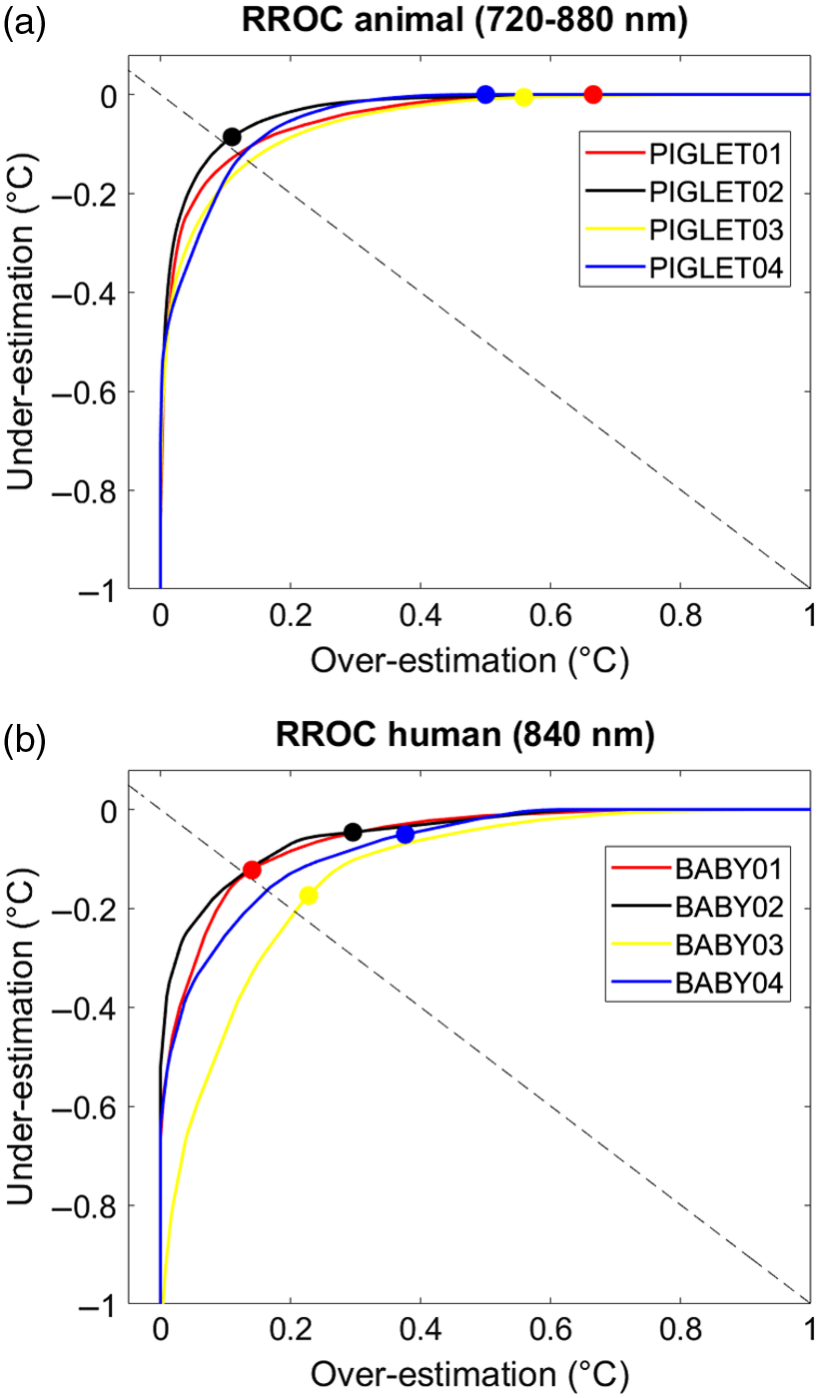
Temperature RROC. RROC curves for each single subject of the animal dataset (wavelength interval 720 to 880 nm with three PCs) and the human dataset (wavelength 840 nm with three PCs). Colored points indicate the MEB per subject that represent over- and underestimation relative to the diagonal (dashed line). See Tables 5 and 6 for statistics.

To graphically illustrate the calibration performance on the single-subject level, we used RROC curves.^43^ The RROC curves allowed for a direct and intuitive visual comparison of the single-subject differences between measured and predicted temperature based on the MEB parameter, which numerically reflects over- or underestimation in temperature prediction. We illustrated each single-subject calibration of the animal and the human dataset for the corresponding best combinations (animal dataset: wavelength interval 720 to 880 nm with three PCs; human dataset: wavelength 840 nm with three PCs; Fig. 7).

**Table 5:** Evaluation metrics. Evaluation metrics for the animal dataset (wavelength interval 720 to 880 nm with three PCs) and the human dataset (wavelength 840 nm with three PCs). Values represent results averaged across for the MAE, MSE, AOC, MEB. See Fig. 6 for illustration.

	Animal dataset	Human dataset
	Three PCs	Three PCs
	720 to 880 nm	840 nm
MAE	0.481	0.358
MSE	0.340	0.200
AOC	0.045	0.079
MEB	0.436±0.283°C	0.162±0.149°C

**Table 6:** Correlation evaluation metrics. Pearson product moment correlation coefficients (r) and p-values between the evaluation metrics, i.e., the MAE, the MSE, the AOC, and the MEB. Correlations were calculated across all subjects of the animal and human datasets.

	MAE	MSE	AOC	MEB
MAE		r=0.994	r=0.029	r=0.925
		p<0.001	p=0.946	p=0.001
MSE	r=0.994		r=0.031	r=0.903
	p<0.001		p=0.942	p<0.001
AOC	r=0.029	r=0.031		r=−0.317
	p=0.946	p=0.942		p=0.444
MEB	r=0.925	r=0.903	r=−0.317	
	p=0.001	p<0.001	p=0.444	

In order to statically compare the evaluation metrics, t-test was performed, i.e., between datasets (animal versus human) and between temperatures (measured versus predicted). Importantly, there was a significant MEB difference when testing both datasets together (animal dataset: t=3.080, p=0.054; human dataset: t=2.170, p=0.118; both datasets: t=7.158, p<0.001; Table 5). This indicated that both the animal and the human dataset exhibited an overestimation of predicted brain tissue temperature compared to the rectally measured temperature. Notably, this was true without differences in the dominance, i.e., the two datasets overlapped in the RROC space.

Otherwise, there were no significant differences between the two datasets regarding the parameters MEB (t=1.709, p=0.138), MAE (t=1.147, p=0.295), MSE (t=1.323, p=0.234), and AOC (t=−1.778, p=0.126), as assessed by independent-samples t-test. This indicated that there were no differences in the overall expected variance of the two datasets based on the applied PLSR. As expected, the evaluation metrics (including both the animal and the human datasets) revealed strong significant linear relationships for MAE and MSE, but not AOC, as assessed using Pearson product–moment correlation (Table 6).

## 5 Discussion

Previous work developed suitable approaches for the calibration of brain temperature prediction using NIRS.^7^^,^^10^^,^^11^ The present analysis provided a first evaluation of broadband continuous-wave NIRS in temperature prediction of brain tissue data derived not only from animals (newborn piglets) but also from human data (newborn infants) undergoing hypothermia treatment. The PLSR calibration applied here allows for the prediction of brain temperature without any knowledge of a reference temperature, which is an important aspect in cases, where measured body temperature is not available. Using this calibration approach, our results showed an overall strong mean predictive power in both the animal ($R^{2}=0.713\pm 0.157$) and the human datasets ($R^{2}=0.798\pm 0.087$). Interestingly, we observed an overall overestimation of the predicted brain temperature compared to the rectally measured temperature of 0.436±0.283°C (animal dataset) and 0.162±0.149°C (human dataset), respectively. We discuss main methodological aspects including the observed overestimation between brain and body temperature, thereby considering interpretation for clinical applications.

### 5.1 Temperature Calibration and Prediction

Based on our findings, we suggest that the accuracy of the temperature prediction depends primarily on three aspects. First, the performance of the calibration method itself (i.e., the PLSR) can affect the prediction and the resulting MEB. For example, a large MEB represents a large absolute difference between measured and predicted temperature (i.e., the amplitude difference between the black dots and the red line in Fig. 6). Second, the amount of noise inherent in the raw data due to temperature-independent signal changes can affect the predicative power in terms of the variance in the $R^{2}$, MAE, and MSE (i.e., the spread of black dots around red line in Fig. 6). Third, the selection of wavelengths employed can affect accuracy. For example, even if a calibration method fits well assuming that absorption at all wavelengths varies linearly with temperature, the errors may be greater at wavelengths where the variation in absorption is comparable to the uncertainty on the absorption values. This variation can be clearly seen in Table 4 in the comparison between the wavelengths 740, 840, and 970 nm. In particular, relative to wavelength 970 nm, the other two bands, 740 and 840 nm, exhibit considerably better temperature prediction. Taken together, these aspects should be considered when selecting a calibration method and interpreting results.

Furthermore, we want to mention some important methodological points regarding our datasets. We observed no significant differences in the accuracy between the two datasets regarding the evaluation metrics MAE, MSE, MEB, and AOC (Table 5), although our data samples were quite small. This could indicate that these datasets were homogenous enough to provide similar accuracies. However, one should consider the following differences between datasets.

First, the wavelength range recorded in the animal dataset (662 to 984 nm, 322 wavelengths) was considerably broader than that in the human dataset (770 to 906 nm, 136 wavelengths). Hence, for the human dataset, we could not assess temperature prediction in more than the 840-nm wavelength. We are currently collecting data in newborn infants with an alternative broadband NIRS instrument with an extended wavelength range that covers the 740 nm, which will allow us to expand the results presented here. We are currently expanding the work in the human subjects by using an alternative broadband NIRS instruments with an extended wavelength range, so then we can investigate the best wavelengths for brain tissue temperature prediction. Second, the animal dataset was recorded during a constant temperature decrease, whereas the human dataset was recorded during a step-by-step temperature increase. The latter resulted in a much smaller amount of data points to be included in the prediction analysis, which may have biased the assessment of the variance in that dataset. Last, the broadband NIRS instruments used to record the animal and human datasets were two different custom-made devices, which may have also contributed to differences in the quality of the data obtained.

### 5.2 Temperature Overestimation

RROC curves were chosen as a graphical tool to compare the performance of the temperature prediction between the individual subjects. We found the RROC curves very suitable for this purpose, because they provided not only a very intuitive illustration of regression performance but also precisely demonstrated differences between measured and predicted temperature (MEB), and allowed for a direct calculation of evaluation metrics (MAE, MSE, AOC).

RROC curves are based on the notion of operating condition, related to cost-sensitive regression with an asymmetric loss function. They, therefore, represent a pendant to the dual positive–negative character in traditional ROC analysis. Asymmetric loss functions often occur in regression models related to real-world problems. Likewise, in our case, there are asymmetric loss functions of the temperature prediction that can be related to clinical application. For example, it is not the same to obtain an overestimation of temperature (i.e., a higher predicted brain temperature compared to measured rectal temperature) versus an underestimation of temperature (i.e., a lower predicted brain temperature compared to measured rectal temperature). This is exactly the problem addressed by asymmetric loss functions and therefore highly relevant for temperature prediction.

In the present analysis, we observed a significant overestimation (as represented by the MEB) in both the animal and the human datasets (Table 5). In other words, we observed that the predicted brain temperature was always higher than the measured rectal temperature in both the animal dataset (mean MEB 0.436±0.283°C) and the human dataset (mean MEB of 0.162±0.149°C; main effect for both datasets: t=7.158, p<0.001; Fig. 7).

There might be several ways to interpret these results. From a methodological point of view, one might assume that systematic measurement errors have contributed to the overestimation. For example, the instrumentation noise might certainly contribute differently to the instrumental error of a given broadband NIRS system that may further depend on a given subject under measurement. From a clinical point of view, one might argue that there exists a physiological reason that explains the overestimation. Previous work in clinical populations (i.e., such as in patients suffering traumatic brain injury) has shown that brain temperature might indeed differ from core body temperature.^44^^–^^47^ These clinical reports should be taken with care, since the existing studies were limited by low sample sizes, varying statistical analysis, and inconsistent measures of brain temperatures (such as epidural, ventricular, cortical, intraventricular, subdural) and core temperatures (rectal, bladder, oesophageal, pulmonary artery). However, these clinical reports may provide some useful assumptions regarding the present analysis. In line with our results, these studies reported that the mean brain–body temperature difference is positive, i.e., indicating that brain temperature is typically higher than body temperature. For example, two studies^14^^,^^48^ examined the differences between brain and rectal temperature during hypothermia. Tokutomi et al.^14^ induced hypothermia in 31 patients to a target (rectal) temperature of 33°C, followed by slow rewarming after 48 to 72 h of hypothermia. Mean brain (subdural) temperature was consistently higher than mean core (rectal) temperature (mean difference 0.5°C, 0.39 to 0.61 CI 95%). Zhang et al.^48^ induced hypothermia in 18 patients to a target (rectal) temperature of 31.5°C, followed by rewarming to 34.9°C for between 1 and 7 days (average 58 h). The mean difference between brain and rectal temperature at 0, 24, and 72 h after therapeutic hypothermia was 0.8°C, 1.1°C, and 1.4°C, respectively. Together, these two studies exemplarily suggested that mean brain temperature was consistently higher than mean rectal temperature at all hypothermia time points. Childs and Lunn^44^ interpreted these findings by pointing out that hypothermia (i.e., in contrast to hyperthermia) is typically associated with overall larger brain–body temperature differences. The authors further reviewed that—whatever the reason for the larger difference reported under hypothermic conditions is (in contrast to hyperthermic conditions)—“when brain temperature falls below 36°C, either by deliberate body cooling or spontaneously as a consequence of brain injury, the dissociation between brain—body temperatures widens by as much as 1.5°C.”

It should be noted that it is so far unclear whether these discrepancies between brain–body temperature measurements under therapeutic temperature interventions are due to effects of the intervention (i.e., no treatment versus hypothermia versus hyperthermia) *per se*, or due to other study-specific aspects, such as the severity and nature of a given clinical population, or rather related to the thermodynamic autoregulation of the brain itself. These aspects are important points to be considered when interpreting our results of the temperature prediction.

### 5.3 Limitations

Last, general methodological limitations of NIRS should be taken into account. As recently reviewed,^49^ in order to measure the hemodynamic response, the NIR light has to pass several layers of biological tissue (i.e., such as the skin, skull, cerebrospinal fluid, etc.) before it is detected in the cortex. Therefore, the thickness of the tissue is an important parameter in determining the depth of cortex penetration and the magnitude of the obtained hemodynamic response. Having said that, however, newborn infants have significantly thinner skin and skull as well as less hair than adults. Together, in infants compared to adults, these aspects therefore result in a reduction of light scattering, an approximately threefold increase in penetration from 3–5 mm to 10–15 mm into the cortex, as well as in a reduction of noise and artifacts due to better contact between skin and optodes. Furthermore, the human dataset was collected within a clinical setting where it was not possible to control the ambient light, the infants were under intensive care so that movement artifacts were common, the optical probes were mounted such as not to interfere with the patient care or cause harm to the infant; therefore, the optode-to-skin contact may have not always been optimal for light transport into the tissue.

Additionally, a potential temperature gradient of the head and brain tissue should be considered. Although, some research has shown that there may be temperature gradients in the form of a horizontal cylinder around the head,^50^ such as during cooling and freezing of the human brain^51^ or heating human hair,^52^ the same research has also reported that such potential gradients vary markedly with body position^50^ and may therefore not be reliable.

**Fig. 8:**
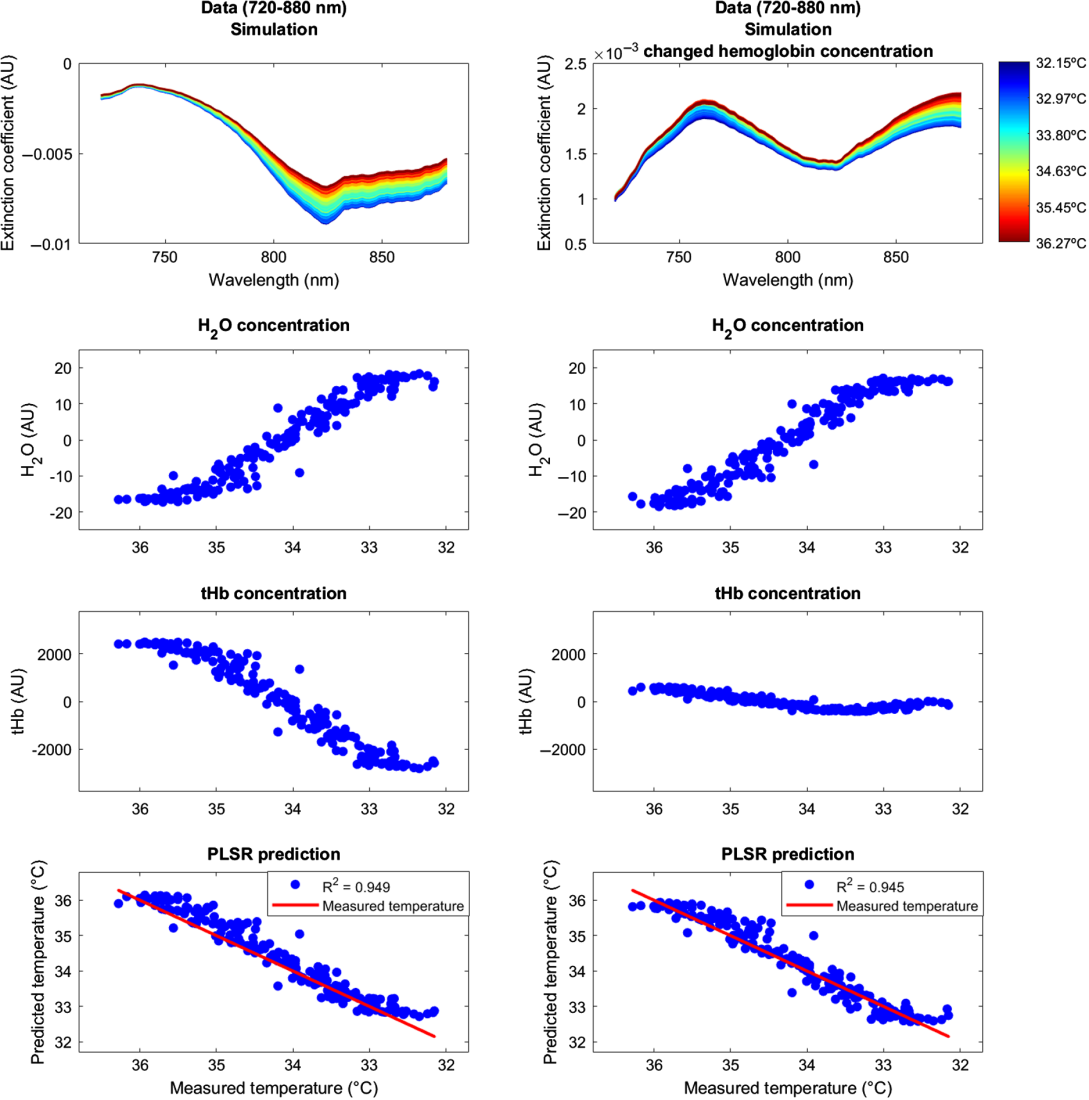
Simulation. We performed a simulation in order to show that the temperature prediction is independent of the hemoglobin concentration. The simulation procedure comprised two steps. (a) In the first step, we simulated a broadband spectrum (720 to 880 nm) using certain arbitrary concentrations of hemoglobin (O2Hb, HHb, with the sum denoted as (b) tHb, (c) water (H2O), and temperature. Using this simulated spectrum, we applied PLSR to predict brain temperature based on a simulated body temperature. (d) In the second step, we then changed only the concentration of O2Hb (with a simple multiplication by 0.1) and simulated the very same spectrum (e) again. This way, we showed that while the tHb concentration changes considerably, (g) the overall temperature prediction was very similar in the two situations (h) and, hence, not affected by the change in O2Hb concentration. Concentrations are shown in arbitrary units (AU).

Last, it should be emphasized that the current data do not provide evidence for temperature prediction in relation to functional activation (see Fig. 8). We clarify that temperature prediction is independent of hemoglobin concentration changes. The reason is because the temperature prediction presented here only uses the temperature coefficient from the pure water absorption spectra in the cross-validated PLSR, and the method should therefore in principle not be dependent on hemoglobin concentration changes. The chromophores of $O_{2}\mathrm{Hb}$ and HHb, which are related to functional activation, are not required for the presented approach and should therefore not impair temperature prediction.

## 6 Conclusion

The present analysis provided a first evaluation of brain tissue temperature prediction using broadband NIRS in human brain tissue. Temperature prediction was best performed using the wavelength interval from 720 to 880 nm with a strong predictive power ($R^{2}>0.7$) and an overall overestimation of NIRS predicted temperature compared to thermometer measured temperature. Although our findings are limited by the small sample size, the present findings may be relevant for the application of brain temperature monitoring in research and clinical settings, such as in critically ill patients, including infants.
